# Rtt109 Prevents Hyper-Amplification of Ribosomal RNA Genes through Histone Modification in Budding Yeast

**DOI:** 10.1371/journal.pgen.1003410

**Published:** 2013-04-04

**Authors:** Satoru Ide, Kimiko Saka, Takehiko Kobayashi

**Affiliations:** 1 National Institute of Genetics, Mishima, Shizuoka, Japan; 2 The Graduate University for Advanced Studies, SOKENDAI, Mishima, Shizuoka, Japan; University of Toronto, Canada

## Abstract

The genes encoding ribosomal RNA are the most abundant in the eukaryotic genome. They reside in tandem repetitive clusters, in some cases totaling hundreds of copies. Due to their repetitive structure, ribosomal RNA genes (rDNA) are easily lost by recombination events within the repeated cluster. We previously identified a unique gene amplification system driven by unequal sister-chromatid recombination during DNA replication. The system compensates for such copy number losses, thus maintaining proper copy number. Here, through a genome-wide screen for genes regulating rDNA copy number, we found that the *rtt109* mutant exhibited a hyper-amplification phenotype (∼3 times greater than the wild-type level). *RTT109* encodes an acetyl transferase that acetylates lysine 56 of histone H3 and which functions in replication-coupled nucleosome assembly. Relative to unequal sister-chromatid recombination-based amplification (∼1 copy/cell division), the rate of the hyper-amplification in the *rtt109* mutant was extremely high (>100 copies/cell division). Cohesin dissociation that promotes unequal sister-chromatid recombination was not observed in this mutant. During hyper-amplification, production level of extra-chromosomal rDNA circles (ERC) by intra-chromosomal recombination in the rDNA was reduced. Interestingly, during amplification, a plasmid containing an rDNA unit integrated into the rDNA as a tandem array. These results support the idea that tandem DNA arrays are produced and incorporated through rolling-circle-type replication. We propose that, in the *rtt109* mutant, rDNA hyper-amplification is caused by uncontrolled rolling-circle-type replication.

## Author Summary

Gene amplification is one of the major strategies used by cells to increase the abundance of gene products. We have been studying amplification of the ribosomal RNA genes cluster, also known as rDNA (ribosomal DNA), in yeast and found that unequal sister-chromatid recombination increased copy number following accidental deleterious recombination events among the repeats. This amplification is highly regulated and ceases when the copy number reaches ∼150. We isolated mutants, including *rtt109*, which have abnormally high copy numbers of rDNA. *RTT109* encodes an acetyl transferase that affects chromatin structure. In the mutant, rolling circle-type amplification that is observed in the early developmental stage in amphibians, oogenesis occured. We speculate that *RTT109* plays a key role in regulating the mode of rDNA amplification. Variation in gene copy number (amplification) has been widely observed in a variety of organisms, contributing to both beneficial adaptation and pathology (e.g., cancer). Our results shed new light on molecular mechanisms of gene amplification.

## Introduction

The ribosome is an abundant macromolecular protein-RNA complex that translates mRNA into protein. In *Saccharomyces cerevisiae*, ribosomal proteins (RP) account for approximately 50% of total protein and ribosomal RNA (RNA) represents approximately 80% of total RNA [1]. While the amount of RP increases during translation from mRNA, the amount of rRNA is dependent on transcription level. To meet this huge biosynthetic demand for rRNA in eukaryotic cells, the rRNA genes are present in hundreds of copies and are transcribed by a highly efficient RNA polymerase, RNA pol I [1]. We recently reported that about half the rDNA genes are transcriptionally silent, but serve as a “foothold” for repair enzymes to maintain the integrity of the rDNA region [2]. Nonetheless, the highly repetitive structure of the rDNA makes it fragile and vulnerable to loss of copies following homologous recombination events within the repeat.

**Figure 1:**
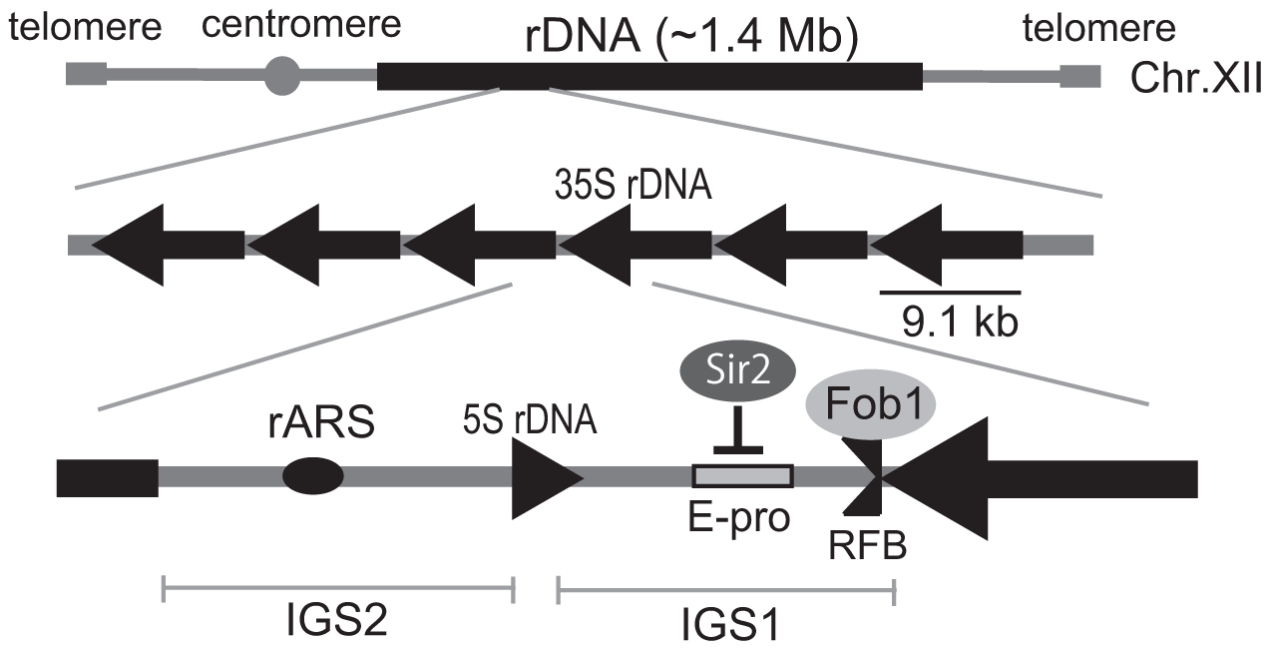
Structure of rDNA region in budding yeast. rARS and RFB are the replication origin and blocking site, respectively. IGS 1 and 2 are intergenic spacers. E-pro is a bidirectional promoter for noncoding transcription. Arrows indicate the direction of transcription of the 5S and 35S rRNA genes. The fork-blocking protein, Fob1p binds to the replication fork blocking site (RFB) in the IGS1, and inhibits the replication fork passing from the left in the figure. Sir2 represses transcription from E-pro.

As an apparent adaptation for a requirement for many copies of the rRNA genes and their simultaneous genetic instability, an rDNA-specific amplification system has evolved in eukaryotic cells. In *Saccharomyces cerevisiae*, the approximately 150 tandemly-repeated rDNA genes are located on chomosome XII (Figure 1A) [3]. One unit of rDNA is 9.1 kb and is composed of a region encoding a pre-35S rRNA, a small 5S rRNA and two intergenic spacer (IGS). The IGS region encodes the key elements involved in the amplification system that maintains rDNA gene copy number [4]. During S phase, progress of the DNA replication fork is blocked at the replication fork barrier sequence (RFB) through the action of the fork-blocking protein, Fob1 [5]. This inhibition induces DNA double-strand breaks (DSBs) and subsequent repair by unequal sister-chromatid recombination which increases rDNA copy number through re-replication of neighbor copies [6]. Moreover, this amplification is promoted by RNA pol II dependent transcription from E-pro, a non-coding bi-directional promoter near the RFB sequence [7]. Transcription from E-pro is proposed to clear cohesin proteins from nearby, which in turn may promote unequal sister-chromatid recombination, because cohesin proteins suppress unequal sister chromatid recombination [7]. In a wild type strain with ∼150 rDNA copies, E-pro transcription is repressed by a histone deacetylase, Sir2. Once rDNA copy number is reduced, Sir2 repression is relieved and amplification by unequal sisiter-chromatid recombination is enhanced. This amplification gradually increases copy number at a rate of ∼ one per cell division until a maximum of about 150 copies is reached [3].

To elucidate the mechanism by which rDNA copy number is regulated, we sought and found mutants in which copy number increased abnormally by screening a genome-wide deletion library consisting of ∼4,800 mutants (OpenBiosystems). Among these mutants, the *rtt109Δ* strain exhibited a prominent phenotype in which rDNA copy number is ∼400 in contrast to ∼150 in the wild type. *RTT109* encodes an acetyltransferase that is stimulated by the histone chaperone Asf1 [8]–[11] to acetylate histone H3 on residue K56. The acetylated histones are incorporated into newly-synthesized DNA to promote replication-coupled nucleosome assembly by chromatin assembly factor 1 (CAF-1) and histone chaperone Rtt106 [12]. It is known that the defect in acetylation affects DNA damage sensitivity, stability of the replication fork, and recombination between sister-chromatids [8], [13]–[16]. Recently, Houseley and Tollervey also independently found this phenotype in the *asf1* and *rtt109* mutants [17].

We analyzed the relationship between histone acetylation and rDNA expansion in depth. We found that mutation of K56 of histone H3 also induced “hyper-amplification” of rDNA. Moreover, the histone deacetylase double mutant, *hst3 hst4*, had an increased rDNA copy number as well. Interestingly, the rate of the hyper-amplification in the *rtt109* mutant was much faster (∼100 copies per cell division), than the ∼1 copy per cell division in wild-type. During hyper-amplification, extra chromosomal rDNA circles (ERC) generated from the rDNA by Fob1-dependent recombination did not accumulate. Instead, integration of plasmids with rDNA sequences was detected. They replicated sequentially and repeatedly during the integration step. These observations suggest that intra-chromosomal recombinational repair of Fob1-driven DSB that produces ERC does not occur. Instead, the data suggest that break-induced replication (BIR) takes place between the DSB end and ERC, resulting in rolling circle replication and hyper-amplification. We conclude that Rtt109 prevents such unusual replication and facilitates completion of recombinational repair with a sister-chromatid after a DNA double-stranded break occurs at the blocking site.

## Results

### Identification of mutants with high rDNA copy number

To elucidate the mechanism which regulates rDNA copy number, we measured the size of rDNA in the ∼4,800 mutants that comprise the yeast deletion library (S288c genetic background) by pulsed-field gel electrophoresis (CHEF, Figure S1). We identified eight mutants with higher-than-usual copy numbers (∼150), and presumed that they were defective in copy number regulation (Table 1). The *rtt109* mutant was among those with the highest rDNA copy number. Chromosome XII of the *rtt109* mutant is bigger than the biggest size maker (3.13 Mb) and sharper than other chromosome XIIs (lane 6 in Figure S1). This indicates that the chromosome XII is too big to be analyzed under these electrophoretic conditions and failed to migrate.

**Table 1:** List of mutants with higher-than-usual rDNA copy numbers.

Gene name	Description from Saccharomyces Genome Database
*POP2*	RNase of the DEDD superfamily, subunit of the Ccr4-Not complex that mediates 3′ to 5′ mRNA deadenylation
*CCR4*	Component of the CCR4-NOT transcriptional complex, which is involved in regulation of gene expression; component of the major cytoplasmic deadenylase, which is involved in mRNA poly(A) tail shortening
*IME4*	Probable mRNA N6-adenosine methyltransferase required for entry into meiosis; transcribed in diploid cells; haploids repress IME4 transcription via production of antisense IME4 transcripts; antisense transcription is repressed in diploids
*RTT109*	Histone acetyltransferase critical for cell survival in the presence of DNA damage during S phase; acetylates H3-K56 and H3-K9; involved in non-homologous end joining and in regulation of Ty1 transposition; interacts physically with Vps75p
*MMS22*	Subunit of an E3 ubiquitin ligase complex involved in replication repair; stabilizes protein components of the replication fork, such as the fork-pausing complex and leading strand polymerase, preventing fork collapse and promoting efficient recovery during replication stress; required for accurate meiotic chromosome segregation
*CTF4*	Chromatin-associated protein, required for sister chromatid cohesion; interacts with DNA polymerase alpha (Pol1p) and may link DNA synthesis to sister chromatid cohesion
*RRN10*	Protein involved in promoting high level transcription of rDNA, subunit of UAF (upstream activation factor) for RNA polymerase I
*DHH1*	Cytoplasmic DExD/H-box helicase, stimulates mRNA decapping, coordinates distinct steps in mRNA function and decay, interacts with both the decapping and deadenylase complexes, may have a role in mRNA export and translation

We measured the size of rDNA in the ∼4,800 mutants from the yeast deletion library (Open Biosystems) by pulsed-field gel electrophoresis. The listed 8 mutants had extremely high rDNA copy numbers. The descriptions are taken from the Saccharomyces Genome Database (http://www.yeastgenome.org/) [46].

**Figure 2:**
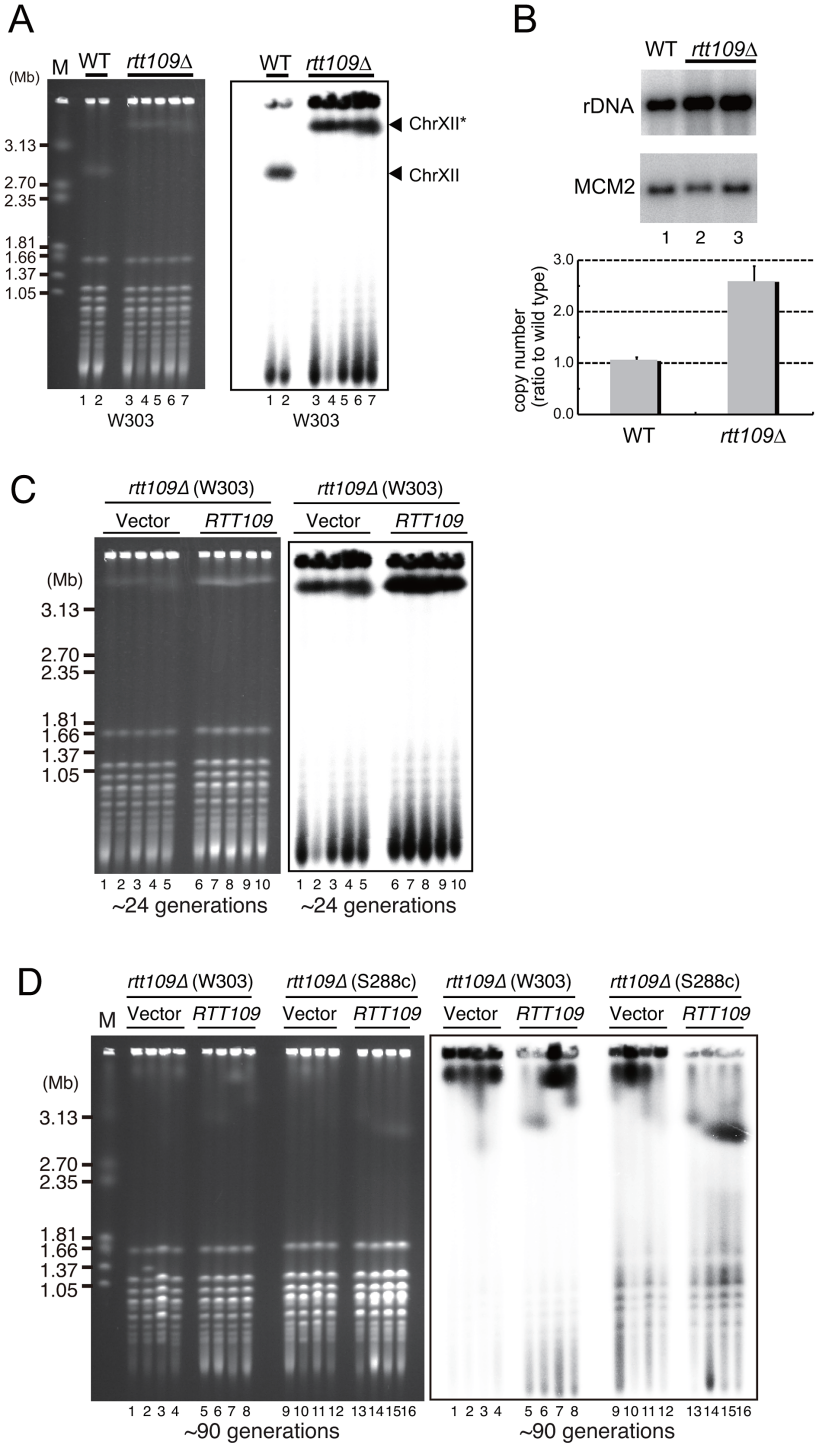
Hyper-amplification of rDNA is induced by deletion of *RTT109*. A) Chromosome XII with rDNA in the wild type and *rtt109* mutant; DNA was analyzed by pulsed-field gel electrophoresis (CHEF), and stained with EtBr (left) and hybridized with rDNA (right). Lanes 1 and 2 are the wild type strains and lanes 3–7 are the *rtt109* mutant in the W303 genetic background. Chromosome XII indicated with the asterisk is longer after hyper-amplification. B) Quantitation of rDNA copy number in the wild type (lane 1) and *rtt109* mutant (lanes 2 and 3) by Southern hybridization. The upper panel shows the rDNA band after *Bgl*II digestion. *MCM2* is the internal control used for normalization. The lower panel indicates quantification of the signal intesity of each band detected by Southern blotting with rDNA and *MCM2* probes, respectively. Values are means of three independent experiments and bars are S.D. values. C, D) *RTT109* restored wild type levels of rDNA copies in the *rtt109* mutant. Chromosome XII was analyzed by CHEF analysis followed by Southern hybridization with an rDNA probe in the *rtt109* mutant at ∼24 generations (C) and at ∼90 generations (D) after introduction of the empty vector (C: lanes 1–5, D: lanes 1–4, 9–12) or plasmid-encoded *RTT109* (C: lanes 6–10, D: 5–8, 13–16). Mutants were constructed in the W303 and S288c genetic backgrounds.

To confirm the “hyper-copy” rDNA phenotype in the *rtt109* mutant, we deleted *RTT109* in a different genetic background (W303) and tested the size of chromosome XII in this mutant by CHEF. As shown in Figure 2A, chromosome XII in the *rtt109* strain was much longer than in the wild type strain. Moreover, we measured rDNA copy number in the *rtt109* mutant based on signal intensities of Southern blots after *Bgl*II digestion (Figure 2B). The copy number was ∼2.5 times greater (∼400 copies) than in the wild type strain (∼150 copies, Figure 2B).

To determine whether hyper-amplification of the rDNA was a direct result of the deletion of *RTT109*, we transformed the *rtt109* mutant with plasmid-encoded *RTT109* and monitored the size of chromosome XII by CHEF. Chromosome XII remained longer up to ∼24 generations following the transformation (Figure 2C, lane 6–10), but decreased in size to that observed in the wild-type strain after ∼90 generations (Figure 2D, lane 5–6 in the W303 background, lane 13–16 in the S288c background), indicating that the hyper-copy rDNA phenotype was due to deletion of *RTT109*.

### Acetylation and deacetylation of histone H3K56 inhibit hyper-amplification

**Figure 3:**
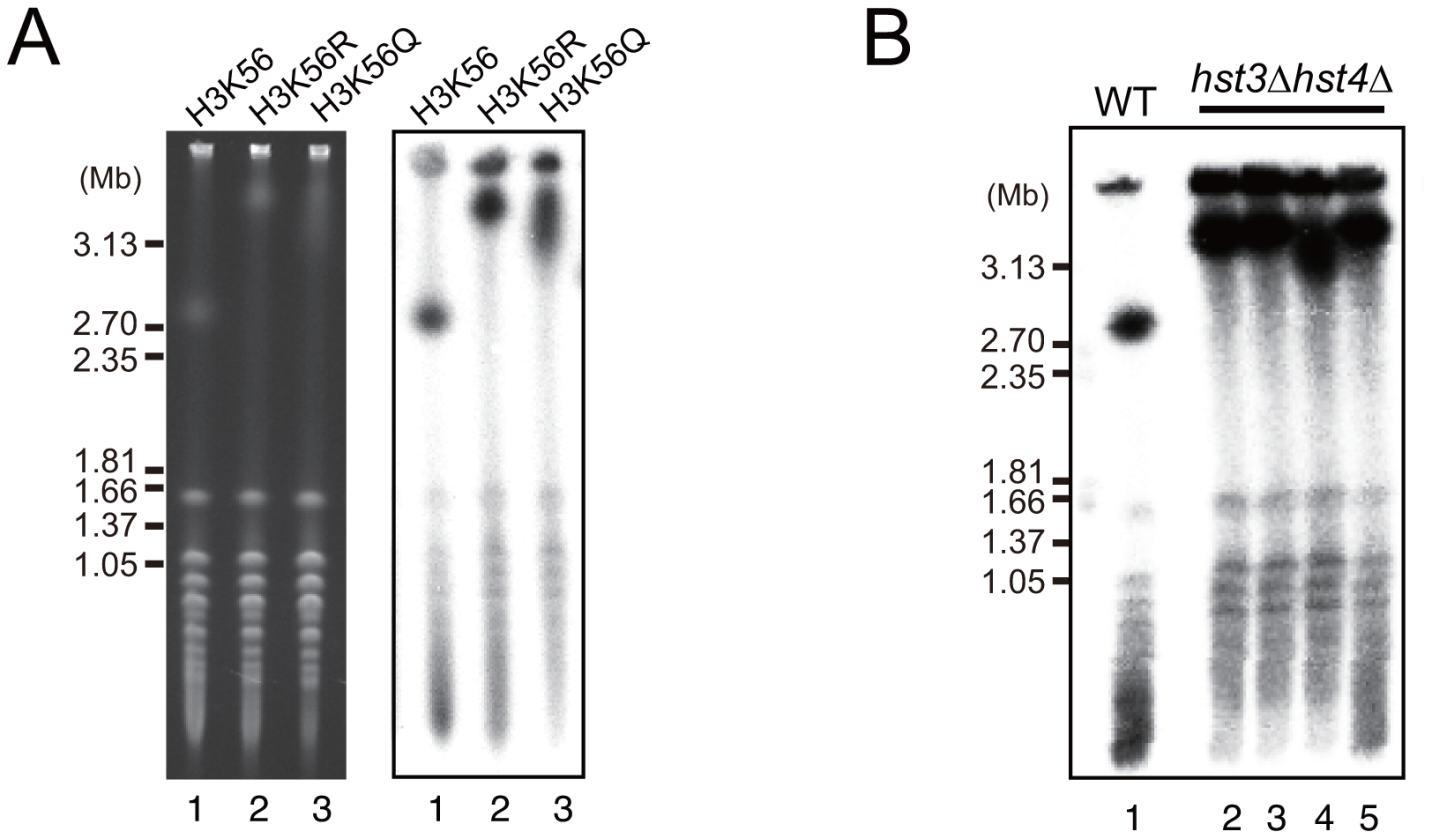
Dysfunction of histone H3K56 acetylation and deacetylation induces hyper-amplification of rDNA copy number. A) Chromosome XII in mutants with amino acid substitutions in histone H3: K56 to R and Q analyzed by CHEF. The left panel shows an EtBr-stained gel and the right panel shows hybridization with an rDNA probe. Lane 1: no substitution (H3K56), lane 2: arginine substituion for lysine (H3K56R), lane 3: glutamine substitution for lysine (H3K56Q). B) Chromosome XII in the *hst3 hst4* double mutant analyzed by CHEF. Lane 1 is the wild type strain as control, and lanes 2–5 are four independent clones of the *hst3 hst4* double mutant strain grown at 23°C.

Rtt109 acetylates the K56 residue of newly-synthesized histone H3 and plays an important role in chromatin remodeling during DNA replication, repair, and recombination [8], [12], [14], [15]. Acetylation of K56 no longer occurs in the *rtt109* mutant [8]–[10]. To determine whether the acetylation activity is related to the hyper-amplification of rDNA, we measured rDNA copy number in the histone H3 mutants, H3K56R (non-acetylated) and H3K56Q (acetylated mimic). As expected, the H3K56R mutant had a longer chromosome XII similar to the *rtt109* mutant (Figure 3A, lane 2), indicating that the hyper-amplification of rDNA occurred by inhibition of acetylation of the histone H3K56 residue. However, unexpectedly, the H3K56Q mutant also had a longer chromosome XII (Figure 3A, lane 3), although the rDNA copy number did not reach the level observed in the histone deacetylase mutant. Moreover, in the W303 genetic background, the double H3K56 histone deacetylase (HDAC) mutant, *hst3 hst4*, in which almost all H3 K56 residues were acetylated, exhibited the hyper-amplification phenotype as well (Figure 3B) [18]. These results indicate that deacetylation of K56 on histone H3 on the sister chromatin also prevents hyper-amplification and maintains proper copy number.

### Hyper-amplification is accelerated by Fob1

**Figure 4:**
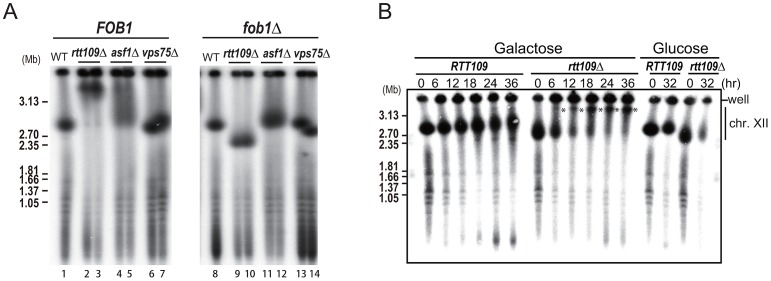
Fob1 promotes hyper-amplification in *rtt109* mutant. A) *FOB1* dependency of hyper-amplification in *rtt109* and related mutants. The size of chromosome XII was analyzed by CHEF. rDNA was detected by Southern hybridization. The left panel shows chromosome XIIs from wild type (lane 1), *rtt109* (lanes 2–3), *asf1* (lanes 4–5), *vps75* (lanes 6–7) strains. The right panel shows chromosome XIIs from wild type (lane 8), *rtt109* (lanes 9–10), *asf1* (lanes 11–12), *vps75* (lanes 13–14) strains in the *fob1* mutant background. B) Time course analysis of hyper-amplification in the *rtt109* mutant. Times after galactose-mediated *FOB1* induction or glucose-mediated *FOB1* repression are indicated. Asterisks show the positions of hyper-amplified chromosome XII containing rDNA. Details are provided in Materials and Methods.

We have previously shown that rDNA amplification from low copy number to wild type levels is dependent on the replication fork blocking activity of Fob1 and subsequent recombination [3]. To determine whether hyper-amplification starting from wild type copy number levels also requires this blocking activity, we deleted *RTT109* in the *fob1* mutant. As shown in Figure 4A (right, lanes 9–10), hyper-amplification in the double mutant was not observed. However, after more than 50 generations, some *rtt109Δ fob1* clones had a longer chromosome XII than the single *fob1* mutant, although they were still shorter than chromosome XII in the *rtt109* single mutant (Figure S2, lanes 15–16). To confirm the contribution of Fob1 to the hyper-amplification phenotype, we monitored amplification rate in the *rtt109 fob1 GAL-FOB1* double mutant over a time course after induction of *FOB1* transcription. Amplification was observed 6 hrs post-induction (Figure 4B, asterisks). Because the doubling time of the mutant we measured was 3.4 hrs, the hyper-amplification phenotype was detectable within ∼2 generations. On the other hand, in cells grown on glucose where *FOB1* was not induced, chromosome XII appeared slightly smeared in the gel, but the longer chromosome XII variant was not detected even after 32 hrs (Figure 4B). Taken together, these observations indicate that Fob1 plays an essential role in the hyper-amplification phenotype. We also tested whether hyper-amplification occurred in mutants belonging to the *RTT109* epistasis group. A histone chaperone, Asf1, functions with Rtt109 to acetylate histone H3K56 [19], [20]. In an *asf1* mutant, the rDNA was also hyper-amplified, although the length of chromosome XII was much shorter than in the *rtt109* mutant (Figure 4A, lane 4–5). Deletion of the other histone chaperone Vps75/Rtt109 complex that acetylates H3 *N*-terminal residues did not promote a hyper-amplification phenotype (Figure 4A, lane 6–7) [21]–[23]. A similar result was reported by Houseley & Tollervey (2011) [17]. In the double *fob1 asf1* mutant, amplification was substantially repressed, while in the *vps75* mutant, no hyper-amplification was observed (Figure 4A, lane 11–14). This is consistent with a previous report that showed that an Rtt109-Asf1, but not an Rtt109-Vps75 complex, acetylated the histone H3/H4 dimer, which is a key event required for genome stability [9].

### The *rtt109* mutation does not affect replication fork stability, non-coding transcription, or cohesin association in the intergenic region

**Figure 5:**
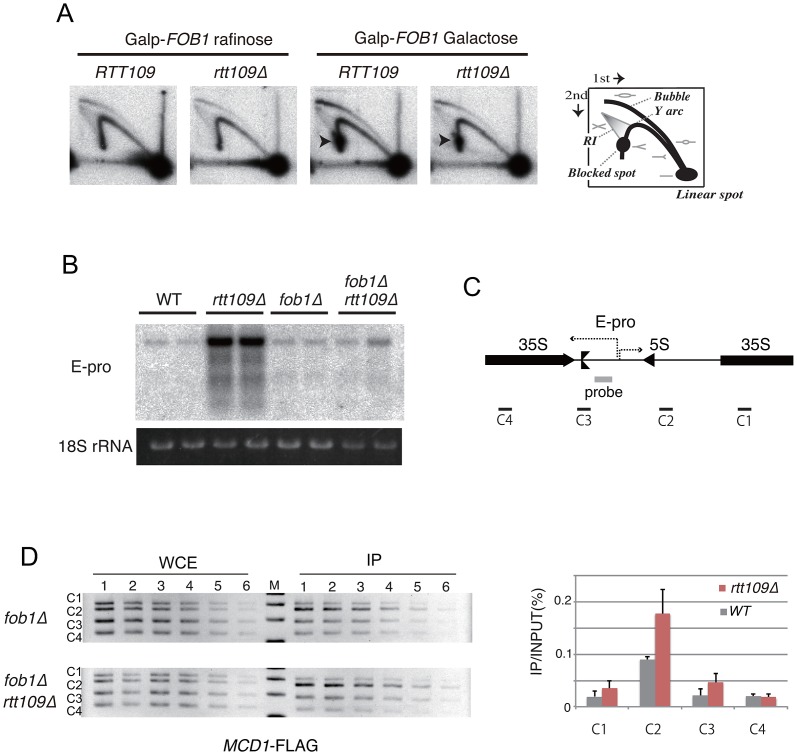
Replication fork stability at RFB, non-coding RNA from IGS and cohesin association in *rtt109* mutant. A) 2D gel electrophoresis to detect replication fork blocking activity at the RFB site with or without Fob1 induction. The scheme is shown in the right side of the figure. RI is recombination intermediates. The wild type (*RTT109*) and *rtt109* mutant with YCplac22-*GAL-FOB1* in the *fob1* defective background were incubated in the medium with raffinose or galactose for 2D gel electrophoresis. See Materials and Methods for details. B, C) E-pro transcription in the *rtt109* and *rtt109 fob1* double mutants. Transcripts from E-pro were detected by northern analysis with the probe positioned on the IGS1 region described in (C) [7]. RNA from two clones of each strain were prepared and analyzed. The 18S rRNA is a loading control. D) ChIP assay for cohesin association to the IGS. The wild type (*fob1* background) and *rtt109* mutant (*fob1* background) both of which carry FLAG-tagged MCD1 were used for the ChIP assay. Four regions within rDNA (C1 to C4 shown in C) were analyzed by PCR. (Left panel) The PCR products from serially-diluted (2-fold) samples were separated in a 2.5% agarose gel and stained with ethidium bromide. (Right panel) Quantification of PCR products. Values of immunoprecipitated DNA (IP) were normalized to total DNA (WCE) and C1 (WT).

Because histone H3K56 acetylation is important for replisome integrity at a stalled replication fork under replication stress [8], [10], we monitored stability of a replication fork arrested at the RFB site in the *rtt109* mutant, in which *FOB1* is induced by galactose as used in Figure 4B, by two-dimensional gel electrophoresis during hyper-amplification. In both the wild type and *rtt109* mutant, the RFB spot on the Y-arc appeared only when *FOB1* was induced (cells grown on galactose). Because the signal intensities were similar (Figure 5A), the fork stability at the RFB appears not to be affected by *RTT109* function. In addition, the amount of recombination intermediates was not increased in the *rtt109* mutant (Figure 5A).

Non-coding transcription from the IGS region (E-pro) is up-regulated during amplification from low copy number rDNA [7]. The transcription reduces association of the cohesin complex, resulting in unequal sister-chromatid recombination [7]. Therefore, we tested whether transcription increased in the *rtt109* mutant. By northern analysis, the RNA increased in the *rtt109* mutant (Figure 5B). However, high level E-pro expression was not observed in the *rtt109 fob1* double mutant (Figure 5B) in which rDNA copy number is maintained at wild type levels (Figure 4A, lanes 9–10). This suggests that the increased rDNA copy number causes the high level of E-pro transcription rather than deletion of *RTT109*. To address this possibility, we measured the level of E-pro in the wild type strain with a higher rDNA copy number constructed by introduction of a plasmid-encoded *RTT109* into the *rtt109* mutant shown in Figure 2C. As the result (Figure S3), the strain with the longer rDNA had a high level of E-pro expression as well as the *rtt109* mutant. Therefore, we conclude that Rtt109 is not involved in repression of E-pro transcription, but that the abnormally increased rDNA copy number leads to a failure to repress transcription. Moreover, we examined the effect of deletion of *RTT109* on the association of cohesin with the IGS region of rDNA by chromatin immunoprecipitation (ChIP) assay. To exclude the indirect effect of increaed rDNA copy number, we compared cohesin (Mcd1) association level between the *rtt109* mutant and the wild type in a *FOB1* defective background. As shown in Figure 5D, we confirmed cohesin association in the IGS in the mutant. Because the association represses unequal sister-chromatid recombination [7], the hyper-amplification in the *rtt109* mutant is most likely not induced by the recombination.

### Rolling circle amplification occurs during hyper-amplification

**Figure 6:**
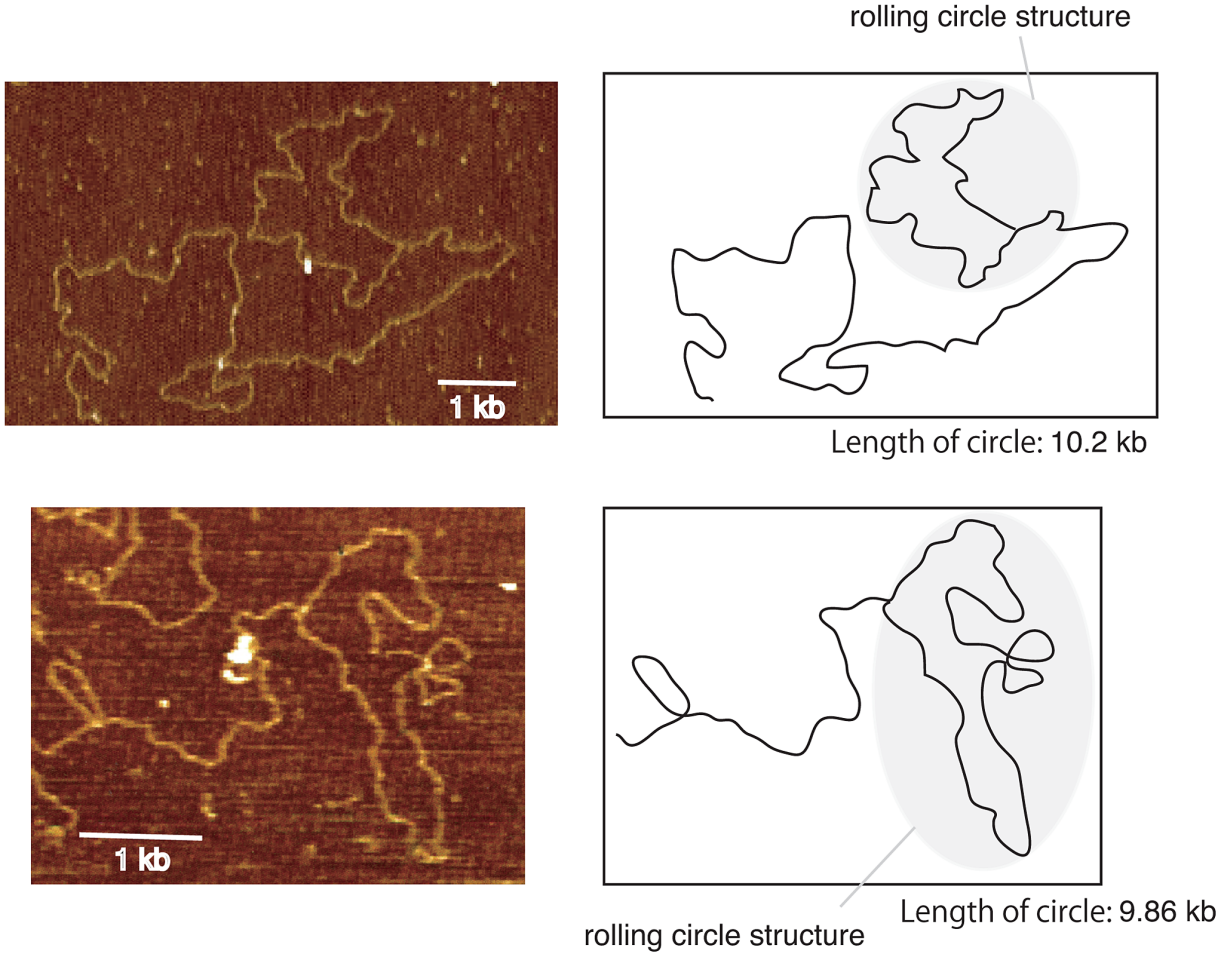
Images of rolling circle structure by atomic force microscopy. DNA was isolated from the well of the CHEF gel for the *rtt109* mutant (12 hr, Figure 4B). By atomic force microscopy, the rolling circle structure with a similar length (8–11 kb) as a unit of rDNA was observed. We observed ∼1% DNA fragments with loop in the *rtt109* mutant. In contrast, in the wild type, such molecules were detected at a frequency of <0.1%. The schemes are shown on the right side of each picture.

**Figure 7:**
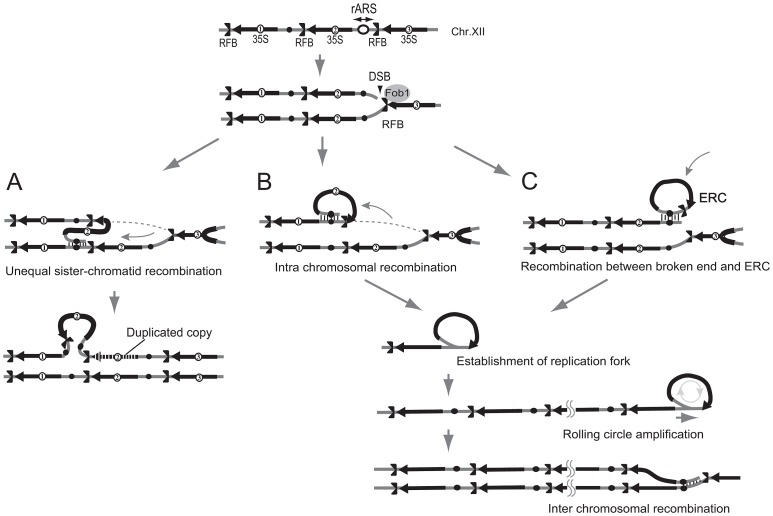
Three models of rDNA amplification. A) Unequal sister-chromatid recombination [3], [7]. In low rDNA copy number, the cohesion complex is displaced by E-pro transcription. The lack of cohesion increases the chance of unequal sister-chromatid recombination occurring in which the misaligned sister-chromatid is selected as the template for repair of the broken end at the RFB. As the result, rDNA copy number changes. B) Rolling circle replication by intra-sister chromatid recombination. The broken end at the RFB recombines via intra-sister chromatid exchange followed by rolling circle replication. C) Alternative mode of rolling circle replication by ERC integration. In this model, ERC is used as a donor for recombination followed by rolling circle replication.

**Figure 8:**
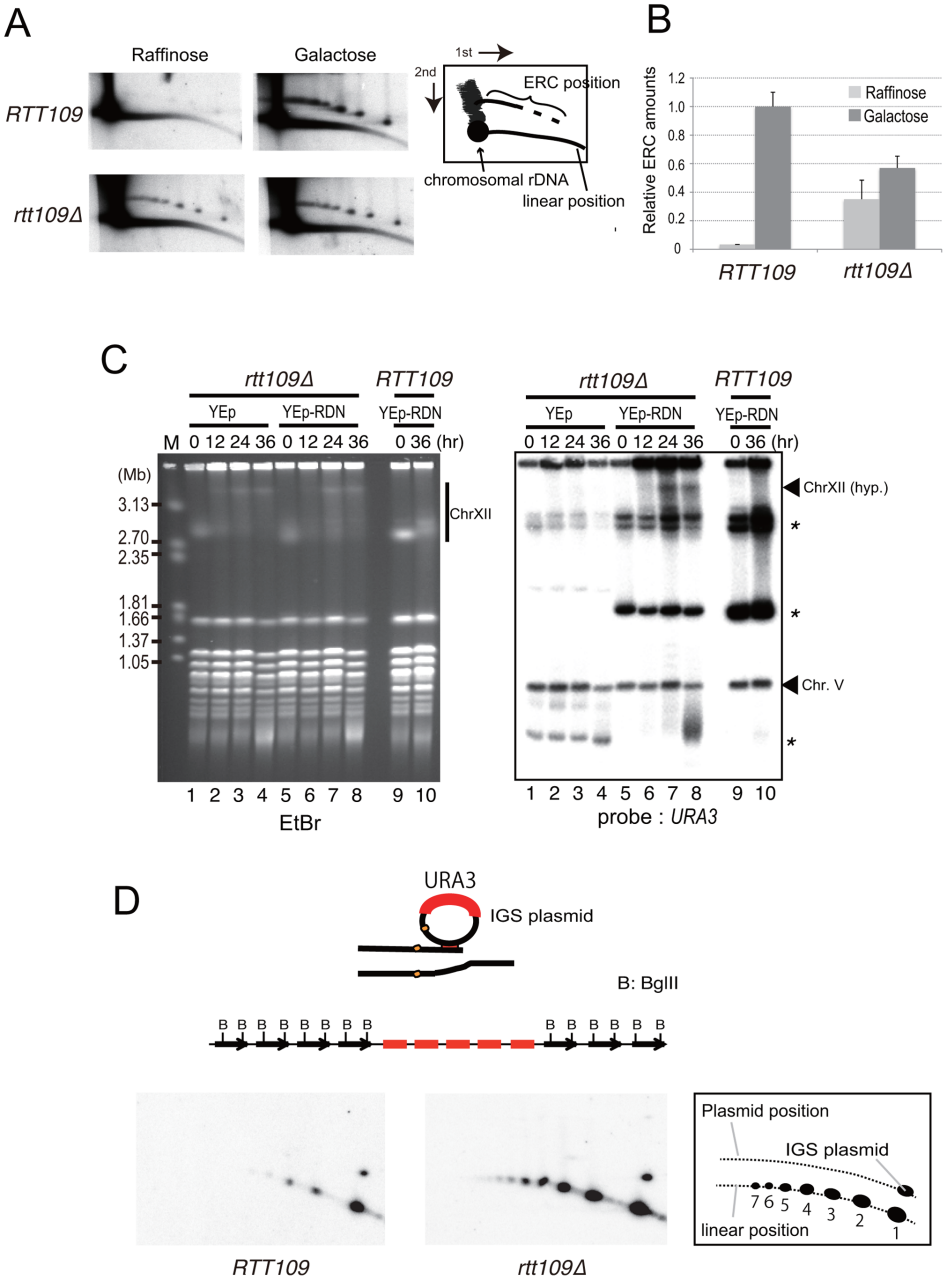
Less ERC is generated and multimeric ERC integrates into rDNA during hyper-amplification. A) 2D gel electrophoresis to detect ERC before (growth on raffinose) and after (growth on galactose) *FOB1* induction. The wild type (*RTT109*) and *rtt109* mutant with YCplac22-*GAL-FOB1* in the *fob1* defective background were incubated as in the experiment to monitor hyper-amplification and were sampled for DNA at time zero (Raffinose) and 9 hr after galactose mediated *FOB1* induction (Galactose). The scheme is shown in the right side of the figure. B) Quantification of ERC before and after *FOB1* induction. Quantification is described in Materials and Methods. C) Integration of plasmid with rDNA into chromosome XII during hyper-amplification; EtBr staining (left) and hybridization with *URA3* as probe (right). Asterisks indicate the positions of the plasmid with and without rDNA (See Figure S4 to confirm the band with asterisks derived from the plasmid). The arrowhead indicates the position of the hyper-amplified band of chromosome XII [ChrXII(hyp.)]. The *rtt109* mutant with an empty vector (lanes 1–4) or with a plasmid containing an rDNA unit (ERC-like plasmid, lanes 5–8) were incubated after *FOB1* induction. As the control, the wild type strain with the ERC-like plasmid was incubated (lanes 9–10, *RTT109*). At the indicated time after Fob1 induction, DNA was prepared for CHEF analysis. Southern hybridization was performed with a *URA3* probe. D) 2D gel electrophoresis to detect the repeats amplified by rolling circle replication. (Upper) A scheme for rolling circle amplification with the IGS plasmid. The amplification forms a cluster of plasmid sequences in the rDNA. Copy number of a cluster is estimated by *Bgl*II digestion. In this case, five copies were amplified. (Lower) Plasmid sequences were detected by a specific probe (amp). The scheme is shown on the right side of the figure. The numbers below the linear position are copy numbers generated by rolling circle amplification.

It appears to be difficult for break-induced unequal sister-chromatid amplification at the standard level to achieve the high rate of amplification observed in the *rtt109* mutant (>100 copies/cell division). As mentioned above, we detected cohesin association that repressed the frequency of unequal sister-chromatid recombination. Rather, rolling circle type amplification appears to occur in such a manner that a large increase in copy number occurs in just a few cell divisions. In fact, the rolling circle structure was observed in the fraction remaining in the agrose gel plug subjected to pulsed-field gel electrophoresis (Figure 6). To initiate rolling circle replication, the broken end at the RFB must recombine with the intra-sister-chromatid (Figure 7B) or with popped-out circular molecules (ERC, extra chromosomal rDNA circle, Figure 7C). In the former case, the recombination usually produces an ERC [4]. Therefore, we monitored ERC formation during hyper-amplification by 2D gel electrophoresis in which the non-linear DNA molecules are separated from genomic DNA. In the wild type control, ERCs were detected in a Fob1-dependent manner as previously described [24], [25]. In contrast, in the *rtt109* mutant, more ERCs were detected than in the wild type strain before *FOB1* induction, while after induction, little increase in ERC level was observed (Figure 8A and 8B). This suggests that Fob1-induced DSB does not change the ERC level, but promotes an alternative pathway, perhaps rolling circle replication.

To test the possibility of a rolling circle amplification for which the ERC serves as a template (Figure 7C), we transformed a *URA3*-plasmid containing an intact rDNA unit (ERC-like plasmid) into the *rtt109 fob1* double mutant and induced *FOB1*. As shown in Figure 8C by Southern hybridization with a *URA3* probe, a signal was detected at the position of the hyper-amplified chromosome XII (ChrXII hyp.) in the *rtt109* mutant. This indicates that the ERC (in this case ERC-like plasmid) was integrated into the rDNA during hyper-amplification in the mutant and the integration may explain little increase of ERC level after Fob1 induction (Figure 8A and 8B). To monitor the status of integration, we performed 2D gel analysis as shown in Figure 8A. For this purpose, the *rtt109* mutant was transformed with another *URA3*-plasmid (IGS-plasmid) containing only the IGS region, after which the DNA was analyzed by digestion with *Bgl*II. The emzyme cuts only chromosomal rDNA units because the IGS region lacks a *Bgl*II recognition site. If rolling circle integration were to occur with the plasmid, tandem arrays of the plasmid sequence would be expected to form, generating long fragments following *Bgl*II digestion (Figure 8D, upper). In contrast, if the integration did not accompany rolling circle replication, a few integrated plasmid sequences would be expected. As shown in Figure 8D lower, signals for the plasmid-specific probe (amp) were observed both at positions corresponding to circular (IGS plasmid) and linear DNAs. Apparently, the copy number for the linear position in the *rtt109* mutant is much higher than in the wild type strain (Figure 8D). This indicates that in the *rtt109* mutant, rolling circle replication occurs more efficiently than in wild type and contributes to hyper-amplification.

### The *rtt109* mutation changes ratio of active to silent copies of rDNA but does not affect transcription rate

**Figure 9:**
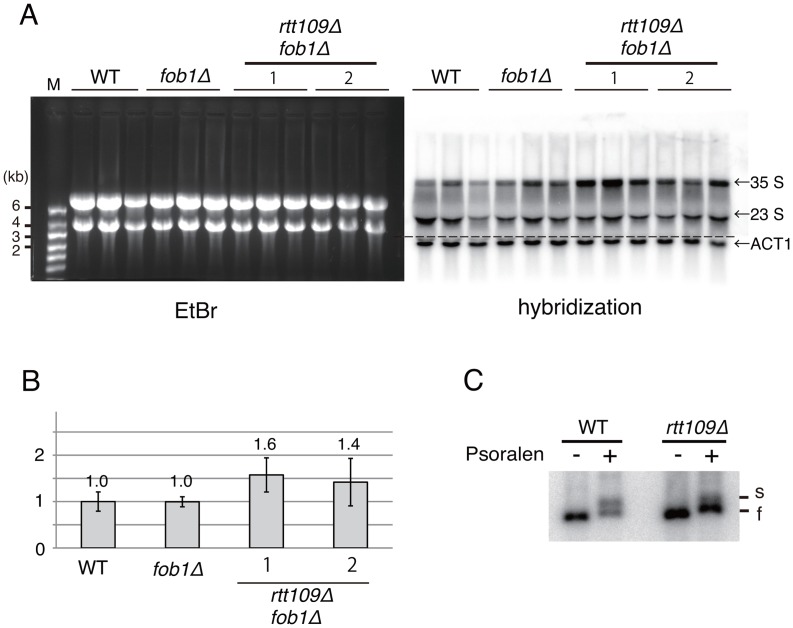
Hyper-amplification of rRNA genes is not a result of transcriptional compensation. A) Nascent rRNA transcription in the *rtt109* mutant. Pre-35S (25S) rRNA and *ACT1* were detected in the *fob1* and *rtt109 fob1* double mutants by northern blotting. (Left) EtBr-stained gel. The bands are mature rRNAs. (Right) Hybridization with pre-35S rRNA-specific oligo probe. *ACT1* was detected with *ACT1* specific probe. B) Quantitation of nascent rRNA transcripts. The pre-35S (25S) rRNA was normalized to *ACT1*. Values relative to the wild type strain. The probe hybridizes with the ETS region of pre-35S rRNA. Therefore, nascent pre-35S and just-processed 25S rRNA with the ETS sequence were detected. Both bands were measured as nascent rRNA transcripts. Values are means of three independent experiments and bars are S.D. values. As for the *rtt109 fob1* double mutants, two sets of experiments were performed. C) The ratio of active (transcribed) to silent (non-transcribed) copies of *rtt109* mutant in the psoralen assay. Genomic DNA from psoralen-treated cells was purified and digested with *Eco*RI. After gel migration, two bands (“s” and “f”) were detected by Southern hybridization with an rDNA probe. “s” and “f” indicate slow and fast moving bands that correspond with active and inactive copies of rDNA, respectively.

It has been reported that some mutants missing transcription factors for rRNA genes (Rrn9, Rrn5, Rrn6, Uaf30), acquired more copies of rDNA to compensate for less efficient transcription [26], [27]. Therefore, we examined whether deletion of *RTT109* decreased transcription of rRNA genes, resulting in compensatory hyper-amplification of rDNA. To exclude indirect effects leading to copy amplification, we compared nascent rRNA in the *rtt109* mutant and in wild type in a *FOB1*-defective background, where hyper-amplification is repressed temporarily. As shown in Figure 9A and 9B, transcript abundance did not decrease in the *rtt109* mutant. Therefore, the amplification is not due to transcriptional compensation.

Further, we examined the transcription status of the rRNA genes in the *rtt109* mutant by psoralen crosslinking [28]. In this assay, because the transcribed (active) rDNA copies have fewer nucleosomes, more psoralen can intercalate into the DNA and retard electrophoretic mobility. As shown in Figure 9C, the intensity of the lower band (silent copies) increased ∼3-fold in the *rtt109* mutant, while that of the upper band (active copies) did not change significantly. This indicates that the hyper-amplification does not increase the number of copies with an open chromatin conformation.

## Discussion

The ribosomal RNA gene is the most abundant and unstable gene in cells. Abundance is maintained in spite of the instability through several mechanisms. Gene amplification after deleterious recombination is one. We found that modification of a histone represses rolling circle replication and maintains wild type rDNA copy number.

Study of the relationship between histone modification, as a phenotype of the *sir2* mutant, and rDNA stability was initiated by Gottlieb and Esposito (1989) [29]. They found that rDNA recombination was greatly enhanced in a *sir2* mutant. Fritze et al. (1997) found that the mutation affected chromatin structure and speculated that it prevented recombination (instability) in the repeat [30]. Later, Sir2 was identified as a histone deacetylase [31]. Subsequently, we found that such alterations of chromatin structure repressed the noncoding promoter (E-pro) whose transcription reduces cohesion association with rDNA, and induces recombination between unequal sister-chromatids, because cohesin proteins hold sister-chromatids together and suppress unequal sister-chromatid recombination [7]. Therefore, one putative mechanism for rDNA maintenance by histone modification is through E-pro regulation. As reported here, however, deletion of *RTT109* alone did not activate E-pro expression when copy number was normal (Figure 5B). Instead, as the result of the hyper-amplification, E-pro transcription was elevated in the *rtt109* mutant. This may be because the copy number is too high to be silenced transcriptionally. It is known that the shorter rDNA also induces a high level of E-pro and induces rDNA amplification [7]. In this case, the activation can be explained by self-regulation of *SIR2* as follows. When rDNA copy number is reduced, a large amount of Sir2 is released from the rDNA and may silence other parts of the genome. In fact, Michell et al. reported that telomere silencing is enhanced in a low rDNA copy strain (2005) [32]. They also showed that *SIR2* itself is a target for repression. Therefore, we propose that autoregulated less *SIR2* expression induces more E-pro transcription to amplify rDNA in a low rDNA copy strain. As copy number increases, more Sir2 is consumed by interaction with rDNA and repression of *SIR2* is reduced. When copy number reaches wild type levels, *SIR2* expression is maximal which represses E-pro resulting in a halt to further amplification. Therefore, in the case of hyper-amplified rDNA, Sir2 levels may be too low to interact with all rDNA copies resulting in a net partial loss of E-pro repression.

Hyper-amplification was observed in the *mms22* mutant which has been shown to interact genetically with *RTT109* (Table 1) [33]. In both *rtt109* and *mms22* mutants, cells become more sensitive to DNA damage during S phase than in wild type. Both Rtt109 and Mms22 are also involved in regulation of homologous recombination induced by replication inhibition [14]–[16], [34]. Our results show that the hyper amplification is accelerated by replication fork-blocking activity in the RFB. Therefore, one explanation is that the blocked fork induces DNA double stranded breaks and that recombinational repair of the broken sister-chromatid can be modulated by these proteins through histone modification. However, as shown in our 2D gels, no significant abnormality was observed in amount of recombination intermediates in the *rtt109* mutant (Figure 5A), e.g., the early steps of recombination (DSB, homologous annealing, strand invasion and formation of Holliday structure). This suggests that Rtt109 and Mms22 function in later steps of the recombination reaction. For example, after formation of the Holliday structure, the donor strand may not separate from the template strand, or, alternatively, replication, such as break-induced replication (BIR) may occur [35]. In case of repetitive sequences such as rDNA, this BIR initiates rolling circle replication which generates an enormous increase in copy number during the cell cycle (Figure 7B).

The pattern of integration of the IGS plasmid into the chromosomal region indicates that rolling circle replication occurs in the *rtt109* mutant (Figure 8D). However, integration of the ERC-like plasmid does not seem to be the sole or main contribution to rDNA amplification. The total signal intensities of both an integrated ERC-like plasmid into chromosome XII [Figure 8C, Chr XII(hyp)] and intermediates of on-going rolling circle replication in the well were ∼15 times greater than that of single copy *URA3* on Chromosome V (Figure 8C; lanes 7–8 in the right pannel, Chr. V). Of course, because both the ERC-like plasmid and native ERC become templates for rolling circle replication, the actual extent of replication is expected to be greater. Intra-chromosomal recombination may also contribute to promote an additional pathway of rolling circle replication. As shown in Figure 7B, the broken end selects the intra-sister chromatid as a recombination template, instead of ERC, and converts into a Break-Induced Replication (BIR) event. Thus, we think that such template switch from sister-chromatid to ERC (results in ERC integration) and BIR conversion (results in less ERC production) reduce Fob1-induced ERC level during hyper-amlification in the *rtt109* mutant (Figure 8A and 8B).

Our rolling circle replication model is supported by other reports concerning Rtt109 function. Wurtele et al. (2012) showed that the histone H3K56R mutation reduced marker loss rate in rDNA [36]. As we showed in Figure 2A, the mutation induced rDNA hyper-amplification which should have reduced loss of rDNA copies. Another study reported that mutated *RTT109* reduced the frequency of unequal recombination between sister chromatids in non-rDNA regions [16]. This is consistent with our observation that cohesin associates with the IGS region of rDNA. This suggests that rDNA amplification by unequal sister-chromatid recombination (Figure 7A) does not seem elevated in the mutant. Houseley and Tollervey (2011) recently reported that hyper-amplification occurred in an *asf1* mutant and proposed a model based on unequal sister chromatid interaction [17]. Because the rate of amplification is much lower in the *asf1* mutant than in the *rtt109* mutant, their model appears plausible. But in the case of the *rtt109* mutant, models other than one based on rolling circle replication would not seem compatible with such a high amplification rate. In addition to the *rtt109* mutant and the histone H3K56R mutant, we found that the double deletion mutant of histone deacetylase that acts on the K56 residue of histone H3, Hst3 Hst4, and the H3K56Q mutant all exhibited a hyper-amplification phenotype. These mutations also increased sensitivity to DNA damage relative to wild type cells [18]. Either an excess or an absence of acetylation on H3K56 of sister chromatids during recombination at the replication fork blocking site leads to rolling-circle amplification of rDNA. While we currently lack a satisfying explanation, the downstream event of the unusual H3K56 modification is important for the completion of recombination at the RFB in order to inhibit BIR-based rolling circle replication.

Rolling circle replication is known to play important roles in cell physiology. The most well known example may be rDNA amplification during amphibian oogenesis [37]. In this stage, extra-chromosomal rolling circle amplification increases copy number up to 2–3 orders of magnitude resulting in a huge increase in rRNAs (and ribosomes) needed for early development [38], [39]. This mechanism of replication is not limited to rDNA but occurs elsewhere in the genome and in some cases, helps cells adapt to various stresses (for review, see [40]). In cancer cells, gene amplification is frequently observed and may be mediated in part by rolling circle replication [41]. Abnormal histone modification has also been reported in malignant tissue [42] consistent with the possibility that gene amplification plays a role in acceleratating tumorigenesis.

## Materials and Methods

### Yeast strains, plasmids, growth conditions for hyper amplification

Yeasts strains used in this study were derived from NOY408-1b in the W303 genetic background and from strain S288c (OpenBiosystems) and are listed in Table S1. The strains were grown at 30°C in YPD medium except for the *hst3 hst4* double mutant that was cultured at 23°C. To monitor rDNA hyper-amplification, the *rtt109* mutant with the plasmid containing galactose-inducible *FOB1* (YCplac22-Gal-*FOB1*) was precultured in synthetic complete medium minus tryptophan with raffinose (SC-trp+raf). The strain was then transferred to SC-trp with galactose instead of raffinose (at time zero) and harvested at the indicated times (0, 6, 12, 18, 24, 36 hr) to prepare genomic DNA in agrose plugs [43]. As a control, the *RTT109* strain was monitored in parallel. Raffinose (1/10 volume of a 10X raffinose stock) and galactose were added just before use. Yeast strains with amino acid substitutions at histone K56 were generated by plasmid shuffling [44].

### Genome-wide screening for yeast mutants with high rDNA copy number

Genomic DNA was purified from ∼4,800 yeast mutants from the genome-wide deletion library (OpenBiosystems) as described [43]. They were then subjected to pulsed-field gel electrophoresis as described below. Gels were stained with ethidium bromide (EtBr). The mutant strains with a longer chromosome XII than the 3.13 Mb size marker (*H. wingei*) were classified as hyper-amplified rDNA mutants (Table 1). We used a wide gel with a long comb (45 teeth) to perform the electrophoresis. Three thin gels were stacked on one another in the electrophoresis tank permitting simultaneous running of ∼130 samples.

### Pulsed-field gel electrophoresis and Southern hybridization

Pulsed-field gel electrophoresis (CHEF) using CHEF Mapper XA (BioRad) and Southern hybridization were performed as described [25], [43]. The conditions were a 300–900 sec pulse time and 100 V for 68 hrs at 14°C in a 0.8% agarose gel.

### 2D gel electrophoresis

To detect replication and recombination intermediates by 2D gel electrophoresis during hyper-amplification, DNA was prepared from cells (YSI204, YSI205) growing in SC-trp+Gal (9 hrs) and SC-trp+Raf, and was digested with *Nhe*I in agarose plugs [43]. rDNA was detected with an rDNA-specific probe. DNA was isolated and digested in agarose plugs.

To detect ERC during hyper-amplification by 2D gel electrophoresis, DNA was isolated from the cells before and 9 hours after release into SC–trp +Gal. To separate large DNAs between 5 and 50 kb by 1D gel electrophoresis, a 0.3% SeaKem Gold Agarose gel (Lonza) was used in TAE at 1 V/cm for 20 hours. After excising each lane above 5 kb from the 1D gel, 2D gel electrophoresis was performed in 1.0% SeaKem LE Agarose gel (Lonza) with EtBr at 6 V/cm for 5 hours at 4°C. The gel was blotted and part of the membrane containing DNAs longer than 7 kb was hybridized with an rDNA-specific probe. The other part of the membrane containing DNAs shorter than 7 kb was probed with *URA3*. The ERC signal was normalized to the *URA3* signal in the each blot. The relative amounts of ERC were determined from three independent experiments as shown in Figure 8B. To detect rolling circle amplification (Figure 8D) by 2D gel electrophoresis, cells with the IGS plasmid, YEplac195 containing the IGS region of rDNA were used. DNA was isolated from cells 48 hrs after release into SC–trp +Gal. Conditions used for 2D gel electrophoresis and induction of hyper-amplification were similar to those described above, except that DNA was digested with *Bgl*II before electrophoresis. *Bgl*II does not cut the plasmid but cuts the chromosomal rDNA, such that only plasmids integrated on the chromosome form linear structures following *Bgl*II digestion. The plasmid sequence was detected using a specific probe (amp).

### Chromatin immunoprecipitation

Chromatin immunoprecipitation was performed as described [6].

### Atomic force microscopy (AFM)

DNA fraction remaining in the agrose gel plug after pulsed-field gel electrophoresis was extracted by crushing. AFM observation was performed as described [45]. During the sample preparation, DNA was sheared into smaller fragments (<∼50 kb). Among these fragments (∼1,500), we looked for the circle with the proper size (8–11 kb). Imaging was carried out with a Didital Instrument MultiMode AFM in the tapping mode. Image J (NIH) was used for measurement of DNA length.

## Supporting Information

Figure S1 An example of the pulsed-field gel electrophoresis screen for strains in which rDNA copy number increased. This is a collection of representative mutants in which rDNA stability was reduced. The gel is stained with EtBr. The largest bands are chromosome XII with rDNA in each lane. WT is the wild type strain. Others are mutants from the deletion library. Lane 6 is the *rtt109* mutant. M is a size marker from *H. wingei*.
(EPS)

Figure S2 Fob1 is required for efficient hyper-amplification. The size of chromosome XII in the *fob1* mutant was monitored for multiple generations after deletion of *RTT109*. *fob1Δ* is a control harboring wild type *RTT109*. Three independent colonies were picked and streaked on YPD to yield single colonies. DNA were then prepared from each and subjected to CHEF analysis. SCI1 is chromosome XII after a single colony isolation (lanes 1–3, 11–13) while SCI3 is a clone obtained after three consecutive single colony isolations (lanes 4–6, 14–16). SCI5 is a clone obtained after five consecutive single colony isolations (lanes 7–9, 17–19).
(EPS)

Figure S3 E-pro transcription in the *rtt109* mutant before and after complementation by the wild type allele. E-pro Transcription in the *rtt109* mutant following complementation with *RTT109*. Transcripts of E-pro were detected by northern analysis in the wild type, *rtt109* mutant and *rtt109* mutant transformed with YCp-*RTT109*. Two clones were analyzed per strain.
(EPS)

Figure S4 Confirmation of extra plasmid-derived bands. EtBr staining (left) and hybridization with *URA3* (right). Data are similar to those shown in Figure 8C except for *rtt109* without the plasmid (lanes 1–2). Electrophoretic conditions were optimized for resolving chromosomes larger than 1 Mb. DNA was prepared for CHEF analysis before (0 hr) and 36 hr after Fob1 induction (36 hr). Chr XII (hyp.) is the hyper-amplified chromosome XII in which *URA3* is integrated and amplified. The arrow head indicates native *URA3* on chromosome V. In the *rtt109* strain without the plasmid, only *URA3* on chromosome V is observed (lane 1–2). This suggests that the other bands are derived from the plasmid carrying *URA3* and may be replication intermediates of plasmid monomers and multimers.
(EPS)

Table S1 Yeast strains and plasmids used in this study.
(XLS)
